# Neutrophils Infiltration in the Tongue Squamous Cell Carcinoma and Its Correlation with CEACAM1 Expression on Tumor Cells

**DOI:** 10.1371/journal.pone.0089991

**Published:** 2014-02-27

**Authors:** Ning Wang, Yuanyong Feng, Qingjie Wang, Shaohua Liu, Lei Xiang, Mingxia Sun, Xiaoying Zhang, Guixiang Liu, Xun Qu, Fengcai Wei

**Affiliations:** 1 Department of Stomatology, Qilu Hospital, and Institute of Stomatology, Shandong University, Jinan, Shandong, China; 2 Department of Pathology, Medical College of Qingdao University, Qingdao, Shandong, China; 3 Department of Oral and Maxillofacial Surgery, the Affiliated Hospital of Qingdao University, Qingdao, Shandong, China; 4 Institute of Basic Medical Sciences, Qilu Hospital, Shandong University, Jinan, Shandong, China; 5 Department of Pathology, Medical College of Shandong University, Jinan, Shandong, China; Sapporo Medical University, Japan

## Abstract

**Objective:**

The present study aimed to explore the clinical significance of neutrophils infiltration and carcinoembryonic antigen related cell adhesion molecule 1 (CEACAM1) expression in the tongue squamous cell carcinoma (TSCC), and to probe the possible relationship between them.

**Materials and Methods:**

Tissue microarray and immunohistochemistry were used to detect neutrophils density and CEACAM1 expression in 74 cases of primary TSCC specimens and 17 cases of corresponding peritumoral tissues. The relationship of CEACAM1 expression and neutrophils density with clinicopathologic parameters and cancer-related survival of TSCC patients were evaluated. The correlation between CEACAM1 expression and neutrophils density was also evaluated. Real-time quantitative transcription polymerase chain reaction (qRT-PCR) was used to explore the possible molecular mechanisms between CEACAM1 expression and neutrophils infiltration.

**Results:**

Immunohistochemistry evaluation revealed that there was more neutrophils infiltration in TSCC tissues than in peritumoral tissues. High neutrophil density was associated with LN metastasis (*P = *0.01), higher clinical stage (*P = *0.037) and tumor recurrence (*P = *0.024). CEACAM1 overexpression was also associated with lymph node metastasis (*P = *0.000) and higher clinical stage (*P = *0.001). Survival analysis revealed that both neutrophils infiltration and CEACAM1 overexpression were associated with poorer cancer-related survival of TSCC patients (*P*<0.05), and neutrophils infiltration was an independent prognostic factor for TSCC (*P*<0.05). Furthermore, overexpression of CEACAM1 was correlated with more neutrophils infiltration in TSCC tissues (*P*<0.01). qRT-PCR results showed that CEACAM1-4L can upregulate the mRNA expression of IL-8 and CXCL-6, which were strong chemotactic factors of neutrophils.

**Conclusion:**

Our results demonstrated that more neutrophils infiltration and overexpression of CEACAM1 were associated with poor clinical outcomes in TSCC tissues. Overexpression of CEACAM1 on tumor cells correlated with more neutrophils infiltration to some extent through upregulating mRNA expression of IL-8 and CXCL-6.

## Introduction

Oral squamous cell carcinoma (OSCC) is among the ten most frequently diagnosed cancers in the world, while the tongue squamous cells carcinoma (TSCC) is the most common type in OSCC [1], [2]. Despite the advancement of diagnosis and treatment, the survival rate of TSCC patients hasn’t been significantly improved during the past decades, mainly due to regional recurrence and lymph node metastasis [3].

Accumulating studies have suggested that inflammation was involved in cancer progression and was the seventh hallmark of cancer [4]. Inflammatory cells include neutrophils, lymphocytes, plasmocytes, macrophages, esinophilic granulocytes etc. Among them, neutrophils represent 50%–70% fraction of total circulating leukocytes [5], [6]. Although neutrophils are traditionally considered antitumoral in the context of their anti-bacterial functions, it is becoming increasingly clear that tumor-associated neutrophils (TANs) play a major role in cancer biology. TANs have been reported to be closely associated with tumor progression and tumor vasculature [7]. The presence of intratumoral neutrophils is a poor prognostic factor in several types of tumors, including localized renal cell carcinoma [8], gastric adenocarcinoma [9], intrahepatic cholangiocarcinoma [10], and hepatocellular carcinoma [11] etc. The study from Trellakis S et al. showed that human head and neck cancer tissues exhibited considerable infiltration by polymorphonuclears (PMNs), and strong infiltration was associated with poorer survival in advanced disease [12]. Whereas, the research of neutrophils infiltration in TSCC tissues has rarely been reported. Further more, how to regulate the inflammatory status of cancers seems particularly important.

Carcinoembryonic antigen-related cell adhesion molecule 1 (CEACAM1), a cell adhesion molecule, belongs to the immunoglobulin superfamily [13] and is expressed on a variety of epithelial, endothelial and hematopoietic cells [14]. CEACAM1 protein has multiple functions, such as regulating cell proliferation, angiogenesis, immunoreaction, tumor invasion and infection of microorganisms etc [13], [15], [16], [17], [18], [19]. The effects of CEACAM1 in neoplasms and inflammatory cells on cells themselves have been elaborated [15], [20], [21], [22]. A recent study showed that cytokine-induced CEACAM1 expression on keratinocytes contributes to a prolonged lifespan of neutrophils [23]. Markel G et al. has also demonstrated that CEACAM1 from melanoma cells can inhibit cytotoxicity of NK cells in a class I MHC-independent way [24]. The above studies implied that CEACAM1 protein in cancerous tissues may have pivotal roles in regulating the inflammation of cancers and so as to influence the cancer biology. However, the research of the effect of CEACAM1 from tumor cells on inflammatory cells remains limited. In this study, we investigated the neutrophils infiltration and CEACAM1 expression in TSCC tissues, analyzed their relationship, and explored the possible roles between them.

## Materials and Methods

### Patients and Specimens

Between 2005 and 2010, a total of 74 patients underwent primary and curative resection for TSCC at the Affiliated Hospital of Qingdao University were selected for the study population and reviewed retrospectively, after obtaining written informed consent from 74 patients. All the 74 patients underwent neck dissection, 34 of which had lymph node metastasis. All the diagnoses were made following the Pathology and Genetics of Head and Neck Tumors of World Health Organization Classification of Tumors [25]. Conventional clinicopathologic parameters, including age, gender, clinical stage, histological grade, tumor size and lymph node metastasis were recorded in Table 1 and Table 2. This study was reviewed and approved by the Institutional Medical Ethics Committee of the Affiliated Hospital of Qingdao University, and written informed consent was acquired from each patient.

**Table 1:** The relationship of CD15+ neutrophils count in TSCC and clinicopathologic parameters.

Clinicopathologic variables	n	CD15+ neutrophils density		*P* value
		Low	High	
**Sex**				0.055
Male	51	21	30	
Female	23	15	8	
**Age**				0.168
≤60 years	41	17	24	
>60 years	33	19	14	
**Clinical stage**				0.037*
I, II	36	22	14	
III, IV	38	14	24	
**Grade**				0.748
G1	40	18	22	
G2	29	15	14	
G3	5	3	2	
**Lymph node metastasis**				0.01*
Yes	34	11	23	
No	40	25	15	
**Tumor extension**				0.682
< 4 cm(T1–T2)	56	28	28	
≥4 cm (T3–T4)	18	8	10	
**Tumor recurrence**				0.024*
Yes	19	5	14	
No	55	31	24	
**Carcinoma**	74	36	38	0.038*
**Adjacent tissues**	17	13	4	

Chi-square test analysis.

*P*<0.05 was considered statistically significant.

**Table 2:** The expression of CEACAM1 on different TSCC groups and its relationship with clinicopathologic features.

Clinicopathologic variables	n	Mean±SD	*P* value
**Sex**			0.358^a^
Male	51	3.373±1.599	
Female	23	3.696±1.663	
**Age**			0.228^a^
≤60 years	41	3.244±1.772	
>60 years	33	3.758±1.370	
**Clinical stage**			0.001^a^ *
I, II	36	2.892±1.430	
III, IV	38	4.027±1.607	
**Grade**			0.742^b^
G1	40	3.375±1.549	
G2	29	3.586±1.701	
G3	5	3.800±1.095	
**Lymph node metastasis**			0.000^a^ *
Yes	34	4.206±1.493	
No	40	2.925±1.385	
**Tumor extension**			0.080^a^
< 4 cm(T1–T2)	56	3.268±1.711	
≥4 cm (T3–T4)	18	4.111±1.079	
**Tumor recurrence**			0.568^a^
Yes	19	3.685±1.024	
No	55	3.416±1.837	
**Carcinoma**	74	3.486±1.217	0.003^a^ *
**Adjacent tissues**	17	2.379±1.133	

a *P* value was estimated by the Mann-Whitney test.

b *P* value was estimated by the Kruskal-Wallis test.

*P<0.05 was considered statistically significant.

### Tissue Microarray and Immunohistochemistry

Tissue microarray (TMA) was constructed as the follows. Firstly, representative areas, away from necrotic and hemorrhagic materials, were premarked in the paraffin-embedded wax blocks by H&E staining. Duplicates of 1-mm-diameter cylinders from tumor center of 74 cases as well as the peritumoral noncancerous squamous epithelial tissue of 17 cases (designated as tumor and peritumoral tissues, respectively) were included in the TMA. Thus, several different TMA blocks were constructed, each containing 42 cylinders. The blocks then were sectioned at a thickness of 4-um thick and placed on slides that were coated with 3-aminopropyltriethoxysilane for immunohistochemistry (IHC).

Infiltration of neutrophils was detected with a mouse anti-CD15 [9] monoclonal antibody (Zhongshan Golden Bridge Biotech, Beijing, China), and CEACAM1 was detected with a mouse monoclonal anti-CEACAM1 antibody (abcam; 29H2, USA). After deparaffinization and rehydration, sections were treated with microwave for heat-induced epitope retrieval in EDTA buffer (95°C). The sections were then washed with PBS and the endogeneous peroxidase activity was blocked with 3% hydrogen peroxide for 10 min. Further, after PBS washing, the sections were incubated with the anti-CEACAM1 (dilution 1∶100) or anti-CD15 (dilution 1∶100) antibodies overnight at 4°C in a humidity chamber. The polymer horseradish peroxidase detection system (ZSGB, CHINA) was applied using DAB for visualization and hematoxylin for nuclear counterstaining. For the negative controls, the primary antibody was replaced with PBS, and the following procedures were the same.

### Evaluation of Immunostaining Parameters

TMAs were evaluated at 200× magnification of light microscopy by two pathologists who were blinded to clinicopathologic data of patients. Slides with debating evaluation were re-evaluated, until a consensus was reached. For CD15+ neutrophils count, positive cells in each 1 mm-diameter cylinder were calculated and presented as mean value of the duplicates (cells/core). The median value was used as cutoff in subsequent analysis. To quantify CEACAM1 expression, proportion and intensity scores were assigned to each specimen. The proportion score represented the estimated proportion of positive tumor cells (0: none, 1: <10%, 2∶10%–33%, 3∶33%–66% and 4: >66%). The intensity score represented the average intensity of the positive tumor cells (0, none; 1, weak; 2, intermediate; and 3, strong). The proportion and intensity scores were then added to obtain a total score, which ranged from 0 to 7 [26]. All specimens were divided into 3 groups for further statistical analyses (Table 3 and Table 4): negative/weak expression, 0–2 points; moderate expression, 3–4 points; strong expression, 5–7 points.

**Table 3:** The relationship of CEACAM1 expression and density of neutrophils.

CEACAM1 expression	CD15+ neutrophils density		Spearman’s rho coefficient test	
	Low	High	*r*	*P* value
**Negative/weak**	21	9	0.782	0.002*
**Moderate**	12	19		
**Strong**	3	10		

*P<0.05 was considered statistically significant.

**Table 4:** Univariate and Multivariate survival analysis in TSCC patients.

Variables	Univariate analysis			Multivariate analysis		
	Relative risk	95% CI	*P*	Hazard ratio	95% CI	*P*
**Gender**						
Male VS female	0.789	0.497–1.315	0.406			
**Age**						
≤60 VS >60	0.674	0.367–1.029	0.332			
**Clinical stage**						
I, II VS III, IV	0.163	0.089–0.274	0.0191*	0.583	0.363–0.991	0.046*
**Differentiation**						
G1 VS G2/G3	0.407	0.342–0.974	0.092			
**LN metastasis**						
No VS Yes	0.239	0.148–0.396	0.026*	0.454	0.315–0.924	0.030*
**Tumor size**						
<4 cm VS ≥4 cm	0.514	0.307–0.832	0.075			
**Tumor recurrence**						
No VS Yes	0.298	0.183–0.549	0.035*	0.627	0.203–1.156	0.061
**CEACAM1 expression**						
Negative/weak VS moderate/strong	0.276	0.161–0.614	0.032*	0.592	0.437–1.072	0.058
**Neutrophils density**						
Low VS high	0.092	0.017–0.458	0.020*	0.325	0.208–0.869	0.019*

CI = Confidence interval.

*P<0.05 was considered statistically significant.

### Cell Culture

Human tongue squamous cell carcinoma cell line Cal-27 were purchased from Culture Collection of Chinese Academy of Science (Shanghai, China) and routinely cultured in Dulbecco’s modified Eagle’s medium (DMEM, Gibco) containing 10% fetal bovine serum (Gibco) at 37°C in a humidified air atmosphere containing 5% CO_2_.

### Construction of CEACAM1-4L and CEACAM1-4S Overexpression Lenti-virus Vector

In humans, 11 different CEACAM1 splice variants have been detected [15]. Individual CEACAM1 isoforms differ with respect to the number of extracellular immunoglobulin-like domains, membrane anchorage and/or the length of their cytoplasmic tail (long or short). CEACAM1-4L and CEACAM1-4S are common isoforms in human tumor tissues [17], [27]. Our IHC results have demonstrated that TSCC have strong CEACAM1 expression. To explore weather TSCC tissues express CEACAM1-4L and CEACAM1-4S, we detected the mRNA expression of them in 12 cases of fresh TSCC tissues. The results revealed that both CEACAM1-4L and CEACAM1-4S have moderate to strong expression in TSCC tissues (Figure S1). Since the expression level of CEACAM1 was very low in Cal-27 cell line, we constructed the CEACAM1-4L and CEACAM1-4S overexpression Lenti-virus (Lv) vectors to simulate the in vivo status. The cDNA sequence of CEACAM1-4L and CEACAM1-4S was a kind gift from Professor John E. Shively [27].

### Transfection of CEACAM1-4L and CEACAM1-4S to Cal-27

A day before transfection, Cal-27 cells, divided into four groups for transfection: CEACAM1-4L-Lv, CEACAM1-4S-Lv, Vector-Lv and blank, were plated at a concentration of 4×10^4^/ml cells in 6-well plates. The second day, when growing to 30–50% confluence, the cells were transfected with the three types of Lenti-viruses in a MOI = 50, premixed with polybrene (5 µg/ml), for 10 h and then the plates were replaced with fresh complete medium without penicillin and streptomycin. After 3 to 4 days, the transfection efficiency can be observed through fluorescent microscope.

### Real-time Quantitative RT-PCR Analysis of CEACAM1-4L and CEACAM1-4S Expression in Different Transfection Groups

4 days after transfection, total RNA was extracted from four groups of cells, using TRIZOL reagent (Invitrogen, USA) according to the manufacturer’s protocol. After DNase I digestion, 2 µg of each RNA samples were reverse transcribed to cDNA using the First Strand cDNA Synthesis kit (TOYOBO, JAPAN). The real-time quantitative transcription polymerase chain reaction (qRT-PCR) was carried out using the SYBR Green qPCR kit (Takara, Japan). The reactions were performed on Mastercycler ep Realplex4 PCR machine (Eppendorf, Germany) with an initial denaturation step at 95 °C for 30 s and then 40 cycles (95°C for 5 s, 60°C for 10 s, 72°C for 15 s), followed by a subsequent standard dissociation protocol. The relative expression values were calculated and normalized to β-actin using the comparative CT method [28]. All reactions were performed in triplicate. The primers for β-actin, CEACAM1-4L and CEACAM1-4S, synthesized by the Shanghai Sangon Biological Engineering Technology & Services Co, are summarized in Table 5.

**Table 5:** Primer sequences and size of PCR products.

	sequences	product size
**β - actin**	5′ - TTGCCGACAGGATGCAGA - 3′	100 bp
	5′ – GCCGATCCACACGGAGTACT - 3′	
**CEACAM1-4L**	5′- ACCCTGTCAAGAGGGAGGAT- 3′	266 bp
	5′- TGAGGGTTTGTGCTCTGTGA - 3′	
**CEACAM1-4S**	5′- ACCCTGTCAAGAGGGAGGAT –3′	234 bp
	5′- GTCCTGAGCTGCCGGTCT –3′	
**IL-8**	5′- CTTGGCAGCCTTCCTGATTTCT - 3′	223 bp
	5′- GTTTTCCTTGGGGTCCAGACAG - 3′	
**CXCL-6**	5′- CCCAAAGCTTGAGTTTCCTGC - 3′	146 bp
	5′- AGTGGTCAAGAGAGGGTTCG - 3′	
**MCP-1**	5′- CTCAGCCAGATGCAATCAATGC - 3′	233 bp
	5′- CCTCAAGTCTTCGGAGTTTGGG - 3′	

### Western Blot Analysis of CEACAM1-4L and CEACAM1-4S Expression

Cellular protein extracts were prepared by homogenization in an ice-cold RIPA lysis (50 mM Tris-HCl, PH 7.4; 150 mM NaCl; 1% Triton X-100; 1% sodium deoxycholate; 0.1% SDS; 2 mM sodium pyrophosphate, 25 mM β-glycerophosphate, 1 mM EDTA; 1 mM Na3VO4, 0.5 µg/ml leupeptin), containing 1 mM phenylmethylsulphonyl fluoride for 60 min. The lysates were centrifuged at 15,000 g for 10 min at 4 °C. Protein concentration was measured using Micro BCA™ Protein Assay kit. Protein was denatured by boiling for 3 min before electrophoresis. 25 µg of each protein sample was subjected to 8% SDS-PAGE, and transferred to Nitroate cellulose membrane. The membrane was blocked with 5% non-fat milk for 1 h, and incubated with monoclonal mouse anti-human CEACAM1 (dilution 1∶500, RD; MAB2244, USA) antibodies for 2 h at room temperature, followed by washing with TBST (TBS containing 0.1% Tween 20) for 5 min five times. After incubation with goat polyclonal anti-mouse HRP-conjugated secondary antibody (Santa Cruz, USA) in TBST for 2 h and washing with TBST for 5 min five times, signals were developed using Immubilon Western Chemiluminescent HRP substrate (Milipore, USA) for 1 min. The images and the intensities of bands were obtained using FluorChem (Alpha Innotech, USA).

### qRT-PCR Analysis of IL-8, CXCL-6 and MCP-1

After verification of CEACAM1 transfection efficiency in mRNA and protein levels, the cDNA was used for qRT- PCR analysis to detect the mRNA expression of IL-8, CXCL-6 and MCP-1 in different groups. The primers information for β-actin, IL-8, CXCL-6 and MCP-1 were listed in Table 5. Amplification products were also separated and visualized on ethidium-bromide stained agarose gels.

### Statistical Analysis

All statistical analyses were performed using the SPSS17.0 (SPSS Inc, Chicago, IL) software package for Windows. The Mann-Whitney test and Kruskal-Wallis test were used to examine the association between CEACAM1 expression and the various clinicopathologic parameters. The Chi-square test was used to examine the correlation of neutrophils infiltration and various clinicopathologic parameters. Correlations of CEACAM1 expression with infiltration of neutrophils were analyzed by Spearman’s rho coefficient test. Survival analysis was estimated by the Kaplan-Meier method and compared by the Log-rank test. Variables that were significant in univariate analysis were subsequently evaluated by multivariate analysis with the Cox proportional hazards regression model. qRT-PCR results were evaluated by student *t* test. *P*<0.05 (two-sided) was considered statistically significant.

## Results

### Patients’ Characters

Patients’ characteristics are listed in Table1. The median age was 59 (range from 32 to 86) and 68. 91% of patients were male. 18 patients had T3/T4 tumors, and 5 patients had high grade tumors. Among all the patients, half of them were in stage III/IV and 34 patients had lymph node metastasis.

### IHC Analysis of Neutrophils Infiltration, CEACAM1 Expression and their Correlation

#### Density of neutrophils in TSCC and peritumoral tissues, and its relationship with clinical pathological features

**Figure 1:**
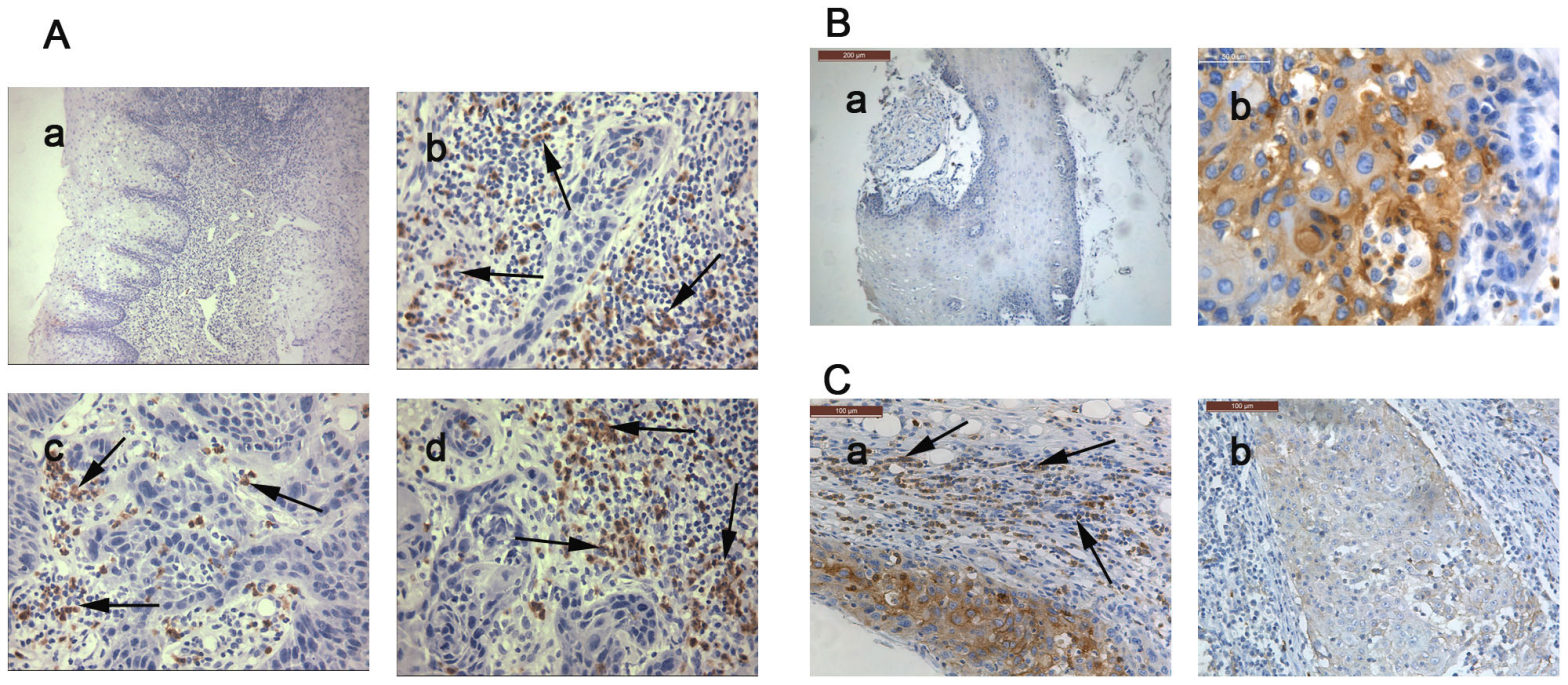
Immunohistochemistry staining results of CD15 and CEACAM1. A: There were more CD15+ neutrophils (black arrow) in TSCC tissues (b, c, d) than in peritumoral tissues (a). In tumors areas, some neutrophils lied in the stroma of tumors (b), some were located within the carcinoma nests (c), and some infiltrated in the borderline of tumor invasion (d). (a: 100×; b, c, d: 400×). B: The expression of CEACAM1 in peritumoral tissues was negative or weak (a). In TSCC tissues, CEACAM1 expression was obviously stronger than that in peritumoral tissues and located mainly in the cytoplasm of tumor cells (b). (a: 100×; b: 400×). C: The relationship of CEACAM1 expression on tumor cells and infiltration of neutrophils. In strong CEACAM1 expression tumors, there were more neutrophils (black arrow) (a); While in negative or weak CEACAM1 expression tumors, there were relatively fewer neutrophils (b). (a, b: 200×, for CEACAM1).

The IHC results of CD15 demonstrated that there were more CD15+ neutrophils in TSCC (Figure 1A b, c, d) than peritumoral tissues (Figure 1A a) (*P = *0.038). The neutrophils infiltrated mainly in intratumoral tissues (Fig. 1A b, c) or in the borderline of tumor invasion (Figure 1A d). In intratumoral areas, the neutrophils were distributed in stroma around the carcinoma nests (Figure 1A b) or within the carcinoma nests (Figure 1A c). In all the 74 cases, 7 cases were lack of any neutrophil (9.59%). The number of CD15+ neutrophils outside the blood vessels was counted in each 1-mm-diameter tissues, which ranged from 1 to 2187/core. The median density was 40.5/core. If the mean number of the duplicates in one case was more than 40.5, it was classified as high density group, otherwise as low density group. In all tumor specimens, the percentage of high CD15+ neutrophils density is 51.35%. Using Chi-square test, we also found that the abundance of CD15+ neutrophils was associated with LN metastasis (*P = *0.01), higher clinical stage (*P = *0.037) and tumor recurrence (*P = *0.024). However, there were no significant differences in gender, age, tumor size and differentiation (Table 1).

### Immunostaining of CEACAM1 in TSCC Tissues and Peritumoral Tissues

We have found there were abundant neutrophils in TSCC tissues and it was associated with poor clinical outcomes. This result was consistent with many studies on other neoplasms [8], [9], [10], [11]. However the mechanisms for this phenomenon of more neutrophils infiltration in cancers remain unclear. Since previous researches have reported that CEACAM1 have pivotal roles to inflammatory cells [23], [29]. We further explored whether there are some relationship between CEACAM1 expression in TSCC tissues and neutrophils infiltration.

Immunohistochemistry results showed that CEACAM1 protein was mainly expressed on cytoplasm or membrane of tumor cells, which was consistent with previous research [30]. CEACAM1 expression in peritumoral tissues was negative or weak (Figure 1B a), whereas its expression was upregulated in cancer tissues (Figure 1B b) compared with peritumoral tissues (*P = *0.003). In cancer tissues, CEACAM1 expression has obvious heterogeneity. Among the 74 cases, 7 were negative, 11 were weakly expressed, 36 were moderately expressed and 20 were strongly expressed. Statistical analysis revealed that strong CEACAM1 expression was associated with lymph node metastasis (*P = *0.000) and higher clinical stage (*P = *0.001) as shown in Table 2.

We also observed that beyond tumor cells, the inflammatory cells in stroma also expressed strong CEACAM1. By comparing with CD15 IHC staining, we found that most of the CEACAM1+ cells were neutrophils and nearly all the neutrophils expressed strong CEACAM1 (Figure S2 a, b, c, d).

### Correlation of CEACAM1 Expression on TSCC with Neutrophils Count

Using Spearman’s rho coefficient test analysis, we found that the density of neutrophils was positively associated with CEACAM1 expression on tumor cells (*P = *0.002, Table 3). That is, in strong CEACAM1 expression group, there were more high density of neutrophils infiltration (76.923%, Figure 1 C a), while in moderate and negative/weak CEACAM1 expression group, there were fewer high density of neutrophils (61.29% and 30% respectively, Figure 1 C b).

### Analysis of the Effects of Neutrophils Infiltration and CEACAM1 Expression on TSCC Patients’ Survival

To assess the effects of neutrophils infiltration and CEACAM1 expression to TSCC patients’ survival, we made a follow up with all the 74 patients. The median follow-up duration was 37 months (range 6–80 months). Within the observation period, there were 17 patients died from cancer.

**Figure 2:**
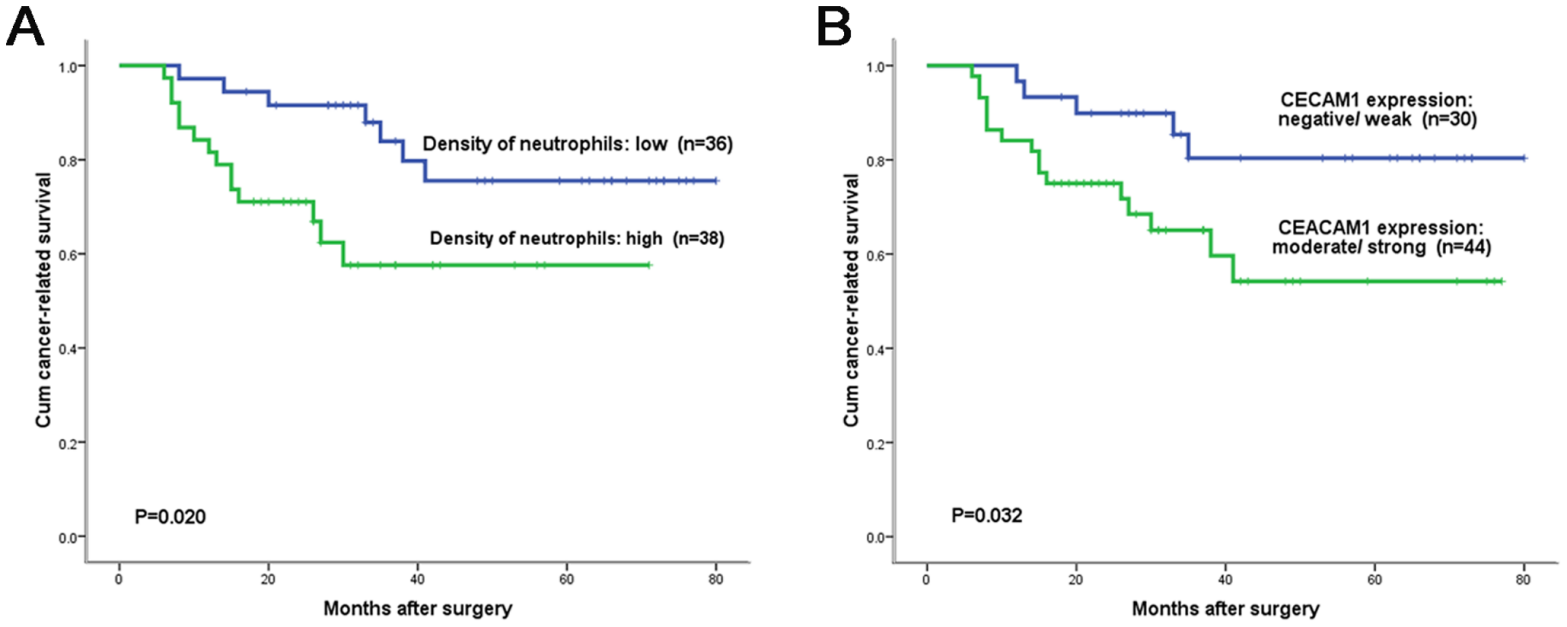
Survival analysis of the effects of CEACAM1 expression and neutrophils density on TSCC pateients. A: The relationship between cumulative cancer-related survival of patients with resectable TSCC and density of neutrophils in tumor tissues. Patients with high density of neutrophils in tumors had significantly reduced cancer-related survival compared to low neutrophils density group (*P = *0.020, Log-rank test). B: The relationship between cumulative cancer-related survival of patients with resectable TSCC and CEACAM1 expression on tumor cells. Patients with moderate or strong expression of CEACAM1 had significantly reduced cancer-related survival compared to negative or weak CEACAM1 expression group (*P = *0.032, Log-rank test).

Kaplan–Meier survival analysis revealed that high-density of CD15+ neutrophils (Figure 2A), strong expression of CEACAM1 (Figure 2B), lymph node metastasis, high clinical stage and tumor recurrence were associated with shorter cancer-related survival of TSCC patients. While tumor sizes (*P = *0.075), tumor differentiation (*P = *0.092), patients’ gender (*P = *0.406) and age (*P = *0.332) had no influence to cancer-related survival. Next, to test whether the above mentioned parameters were independent prognostic factors for TSCC patients’ survival, we performed a multivariate survival analysis using the Cox proportional hazard model, in which those parameters associated with cancer-related survival in the univariate survival analysis were included in the multivariate survival analysis model. The results showed that high density of CD15+ neutrophils, lymph node metastasis and high clinical stage were independent prognostic factors for TSCC patients (Table 4).

### CEACAM1 can Attract more Neutrophils to Tumor Sites Through Upregulating the mRNA Expression of IL-8 and CXCL-6

**Figure 3:**
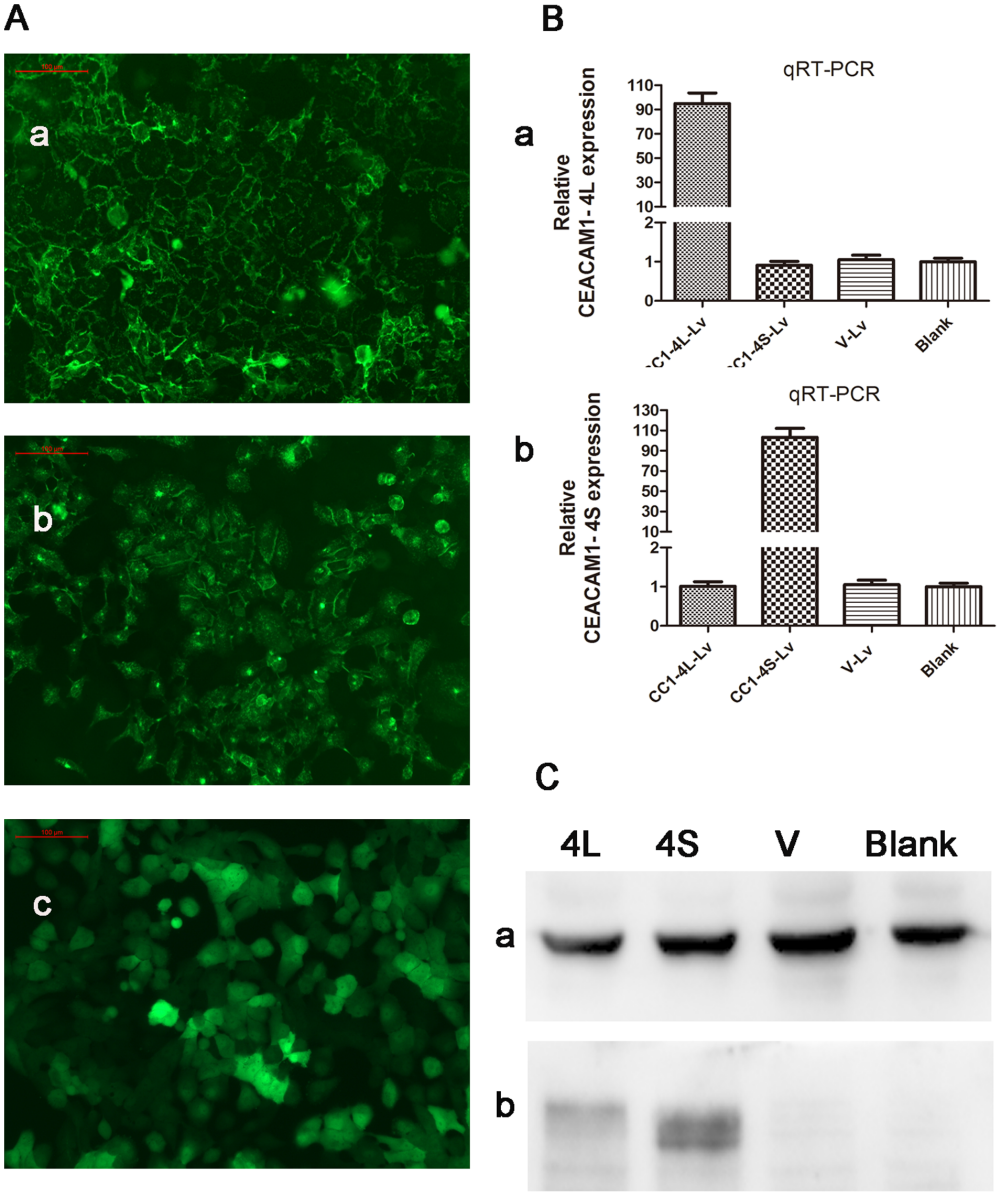
Verification of CEACAM1 overexpression Lv transfecion to Cal-27. A: Fluorescence microscopic observation of Lv transfection efficientcy. a: CC1-4L-Lv group; b: CC1-4S-Lv group; c: Vector-Lv group. (a, b, c 200×). B: qRT- PCR results of Lv transfection. The results showed that CEACAM1-4L mRNA expression was obviously higher in CC1-4L-Lv transfection group than the other three groups (a), and CEACAM1-4S mRNA expression was the same as 4L (b). C: Western blot results for Lv transfection. a: β-actin; b: CEACAM1-4L (the above band) and -4S (the lower band). Results showed that CEACAM1-4L protein expression was obviously stronger in CC1-4L-Lv transfection group than the other three groups. CEACAM1-4S protein expression was the same as 4L. (Note: CEACAM1-4L protein was slightly bigger than CEACAM1-4S).

Since Cal-27 cell line expressed low level of CEACAM1, CEACAM1-4L and CEACAM1-4S overexpression LV vectors were constructed and transfected to Cal-27 to simulate the in vivo environment. The transfection efficiency can be observed through fluorescence microscope (Figure 3A a, b, c) after transfection for 3 or 4 days. The expression of CEACAM1-4L and -4S was obviously elevated in CEACAM-4L and -4S transfection group respectively compared with empty vector transfection group and blank group through qRT-PCR (Figure 3B) and western blot analysis (Figure 3C).

**Figure 4:**
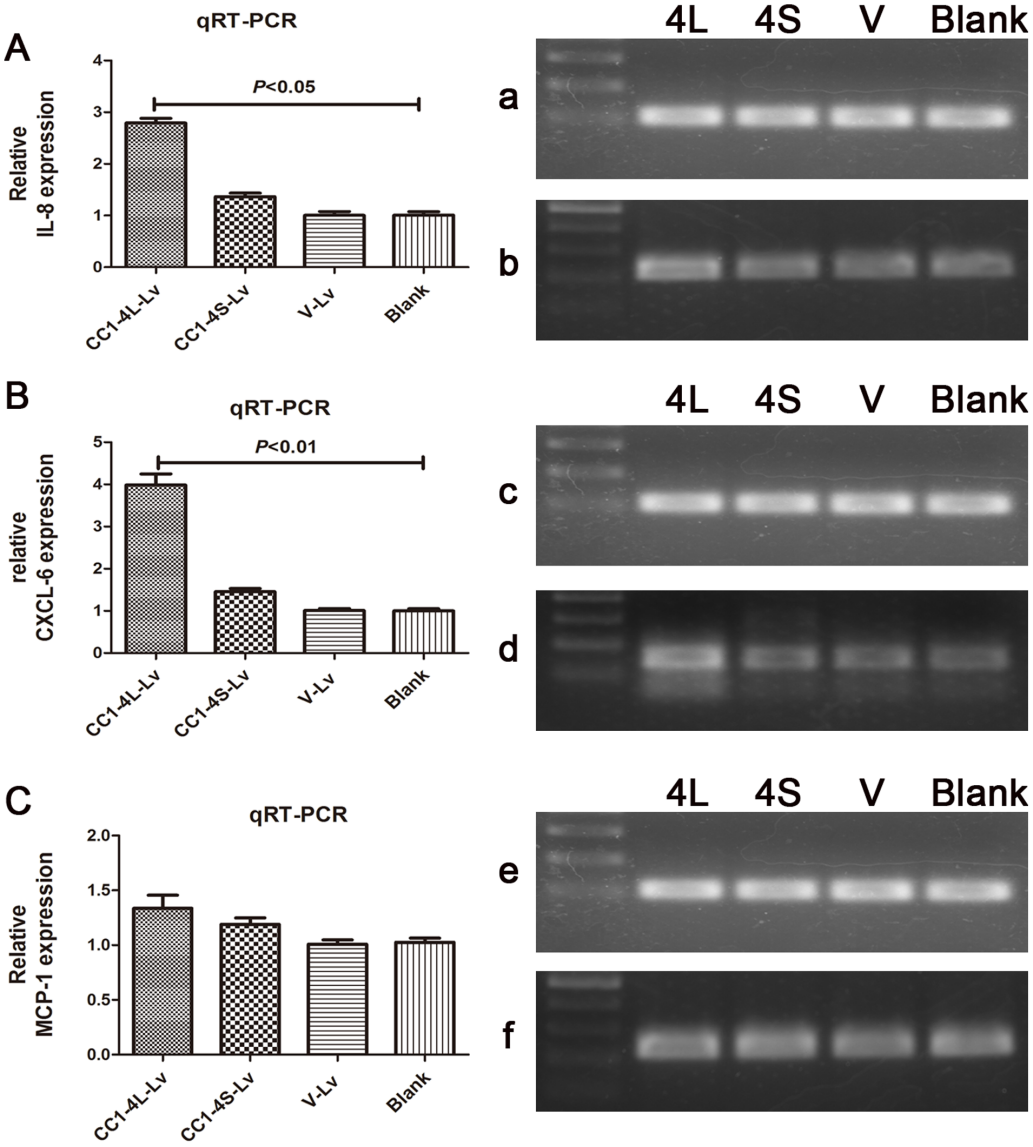
mRNA expression of IL-8, CXCL-6 and MCP-1 in four groups using qRT-PCR. A: The relative mRNA expression of IL-8 was significantly higher in CC1-4L-Lv group than in V-Lv and Blank group (*P*<0.05), while there was no obvious alteration in CC1-4S-Lv group. B: The relative mRNA expression of CXCL-6 was significantly higher in CC1-4L-Lv group than in V-Lv and Blank group (*P*<0.01), while there was no obvious alteration in CC1-4S-Lv group. C: The relative mRNA expression of MCP-1 had no significant differences between four groups. Note: Amplification products of qRT-PCR were also separated and visualized on ethidium-bromide stained agarose gels (corresponding right parts). a, c, e: β-actin; b: IL-8; d: CXCL-6; f: MCP-1.

Because the IHC results demonstrated that CEACAM1 expression was positively related to CD15+ neutrophils count, we speculated that there might be some relationship between them. We explored the possible effect of the overexpressed CEACAM1 in tumor cells to neutrophils infiltration. Using qRT-PCR, we found that CEACAM1-4L overexpression could upregulate the mRNA expression of IL-8 (Figure 4 A) and CXCL-6 (Figure 4 B) (*P*<0.05 and *P*<0.01 respectively), but not MCP-1 (Figure 4 C) in Cal-27 cell line. While CEACAM1-4S had no influence to them all (Figure 4 A, B, C). Previous studies have found that CXCL-6 and IL-8 are important chemokines for neutrophils [31], [32], [33], [34], [35]. Our results revealed that overexpression of CEACAM1 on tumor cells may promote neutrophils infiltration.

## Discussion

More and more evidences demonstrated that inflammation was involved in tumor initiation and progression [4], [36]. Neutrophils account for the largest population of the whole circulating leukocytes and play an established role in host defense of invading microorganisms [36]. Increasing studies have showed that neutrophils played an important role in cancer biology besides its traditional anti-bacterial function [37]. In a series of tumors, more neutrophils infiltration predicted a poorer prognosis and associated with a shorter overall survival [8], [9], [10], [11], [35], [38]. However, the reports of neutrophils infiltration in TSCC tissues were rare and the regulation mechanisms were unclear.

CEACAM1, a transmembrane glycoprotein, is a member of immunoglobulin superfamily and belongs to the CEA family [14]. CEACAM1 is expressed on a variety of cells and has pleiotropic functions. Researches have demonstrated that CEACAM1 has important regulatory roles to neutrophils’ function and apoptosis [23], [29]. In the current study, we explored the expression of CEACAM1 and the count of neutrophils in TSCC tissues, their relationship and the possible effects of CEACAM1 from tumor cells on neutrophils.

Firstly, our study showed that the CD15+ neutrophils infiltration was abundant in TSCC tissues compared with peritumoral tissues (Figure 1 A) and had great heterogeneity. In LN metastasis TSCC group, there were significantly more CD15+ neutrophils than without LN metastasis group (Table 1). The same phenomenon of more neutrophils infiltration can also be observed in clinical stage III/IV groups and tumor recurrence group (Table 1). This indicated that abundant infiltration of CD15+ neutrophils predicted poor clinical outcomes. What’s more, survival analysis revealed that high density of CD15+ neutrophils infiltration was an independent prognostic factor for TSCC patients. These results were in line with observations from several other groups in other tumors [8], [9], [10], [11]. Studies have showed that neutrophils promoted angiogenesis during the early stages of carcinogenesis [39]. Neutrophils could regulate the oncogene-induced keratinocyte hyperproliferation during skin tumorigenesis [36] and prevent apoptosis of tumor cells in the lung microenviroment [40]. Neutrophils could also assist tumor cells extravasation during the metastatic process through secreting a series of enzymes [41] and serve as a carrier to assist tumor cell transendothelial migration [42], [43]. On the other hand, neutrophils can inhibit T-cell effector functions and proliferation through the secretion of stored arginase 1 which degrades extracellular arginine, a factor needed for the proper activity of T-cells [44], [45]. These researches indicated that the neutrophils can promote tumor progression and metastasis through a series of mechanisms. However the reason for this phenomenon of abundant neutrophils infiltration in tumors remains unclear.

Since CEACAM1 has pivotal function to inflammatory cells, we explored the expression and possible roles of CEACAM1 in TSCC tissues. Our result showed that CEACAM1 expression in cancer tissues was higher than peritumoral eptithelial tissues (Figure 1B). CEACAM1 was expressed at a higher level in LN metastasis group than without LN metastasis group, and its expression was also associated with higher clinical stage (Table 2). Survival analysis revealed that overexpression of CEACAM1 was correlated with shorter cancer-related survival, but was not an independent prognostic factor for TSCC patients. These results implied that CEACAM1 overexpression in TSCC was associated with poorer clinical outcomes. This observation was consistent with previous studies on non-small cell lung cancer [46], malignant melanoma [47], thyroid carcinoma [20], hepatocellular carcinoma [21] and colorectal cancer [18], [48] etc. A series of studies have demonstrated that CEACAM1 can promote tumor progression and metastasis through many ways. For example, studies on oral squamous cell carcinoma [30] and non-small cell lung cancers [22] showed that CEACAM1 overexpression were associated with angiogenesis. Alireza Ebrahimnejad et al have demonstrated that CEACAM1 enhanced invasion and migration of melanocytic and melanoma cells [17]. More recently, the study of Zhangguo Chen et al showed that CEACAM1 dampens antitumor immunity by down-regulating NKG2D ligand expression on tumor cells [16]. These researches implied that CEACAM1 overexpression in TSCC may also promote tumor progression and so as to be correlated with poor clinical outcomes.

More importantly, our results demonstrated that in CEACAM1 strong expression group, there were highest density of neutrophils (Figure 1 C a), while in CEACAM1 weak and negative expression cases, there were fewer neutrophil infiltration (Figure 1 C b). Spearman’s rho coefficient test result showed that CEACAM1 expression was positively related to the density of CD15+ neutrophils in TSCC tissues (Table 3). These results implied that CEACAM1 overexpression in TSCC was associated with neutrophils infiltration and location. To explore the possible effects of CEACAM1 in tumor cells to neutrophils, we detected the mRNA expression of IL-8, CXCL-6 and MCP-1 in different CEACAM1 transfection groups (Figure 4). The results showed that overexpression of CEACAM1-4L could upregulate IL-8 and CXCL-6 mRNA expression, which are strong chemokines that are involved in neutrophils recruitment and migration [34], [35]. This indicated that CEACAM1 may attract more neutrophils to the tumor sites through upregulating neutrophil chemokines’ expression. Our study is in line with previous study on endothelial cells, which showed that overexpression of CEACAM1 on human microvascular endothelial cells can lead to upregulation of IL-8 [49]. Study from Lievin-Le Moal V et al. showed that apical expression of full-length hCEACAM1- 4L renders kidney cells responsive to LPS leading to TLR4-dependent Erk1/2 and p38 MAPK signaling [50]. While the p38 MAPK signaling can regulate the production of a series of cytokines in the tumor microenvironment, including CXCL-6 and IL-8 [51], [52]. These studies implied that overexpression of CEACAM1 on TSCC may upregulate CXCL-6 and IL-8 through p38 MAPK signaling, and so as to attract more neutrophils to the tumor sites. But these need to be further studied in future. We also found that nearly all the neutrophils expressed strong CEACAM1 (Figure S2). Rahmoun M et al. have demonstrated that cytokine-induced CEACAM1 expression on keratinocytes contributes to a prolonged lifespan of neutrophils. Study form Singer BB et al. showed that CEACAM1 can mediate delay of apoptosis in granulocytes [29]. These researches demonstrated that both exogenous and endogenous CEACAM1 can prolong neutrophils’ lifespan. This may also explain the correlation of CEACAM1 expression on tumor cells and neutrophils density. Since neutrophils could also promote tumor progression through a series of ways [34], [39], [53], [54], [55]. We speculated that the correlation between CEACAM1 expression and neutrophils infiltration may represent another cause for the poor clinical outcomes of CEACAM1 overexpression in cancers. In conclusion, our study demonstrated here that both CEACAM1 overexpression and abundance of neutrophils were associated with poor clinical outcomes in TSCC patients. CEACAM1 expression on tumor cells was associated with more neutrophils infiltration. The overexpressed CEACAM1 may attract more neutrophils to tumor sites through up-regulating IL-8 and CXCL-6 expression.

## Supporting Information

Figure S1 **Semiquantitative RT-PCR analysis of CEACAM1-4L and CEACAM1-4L in TSCC tissues.** The results showed that both CEACAM1-4L and CEACAM1-4S were moderately or strongly expressed in TSCC tissues. 1–6 represented 6 cases of the fresh TSCC tissues.
(TIF)

Figure S2 **CEACAM1 and CD15 immunohistochemical staining for neutrophils in SCCOT tissues.** The results showed that most of the CEACAM1 positive inflammatory cells were neutrophils and nearly all the neutrophils expressed strong CEACAM1. a, c: CEACAM1 staining; b, d: CD15 staining. (a, b, c, d 200×).
(TIF)
