# Supplementary figures and images for: Neutrophils Infiltration in the Tongue Squamous Cell Carcinoma and Its Correlation with CEACAM1 Expression on Tumor Cells
Source: PLoS One. 2014 Feb 27;9(2):e89991. doi: 10.1371/journal.pone.0089991 (PMC3937421; doi:10.1371/journal.pone.0089991)

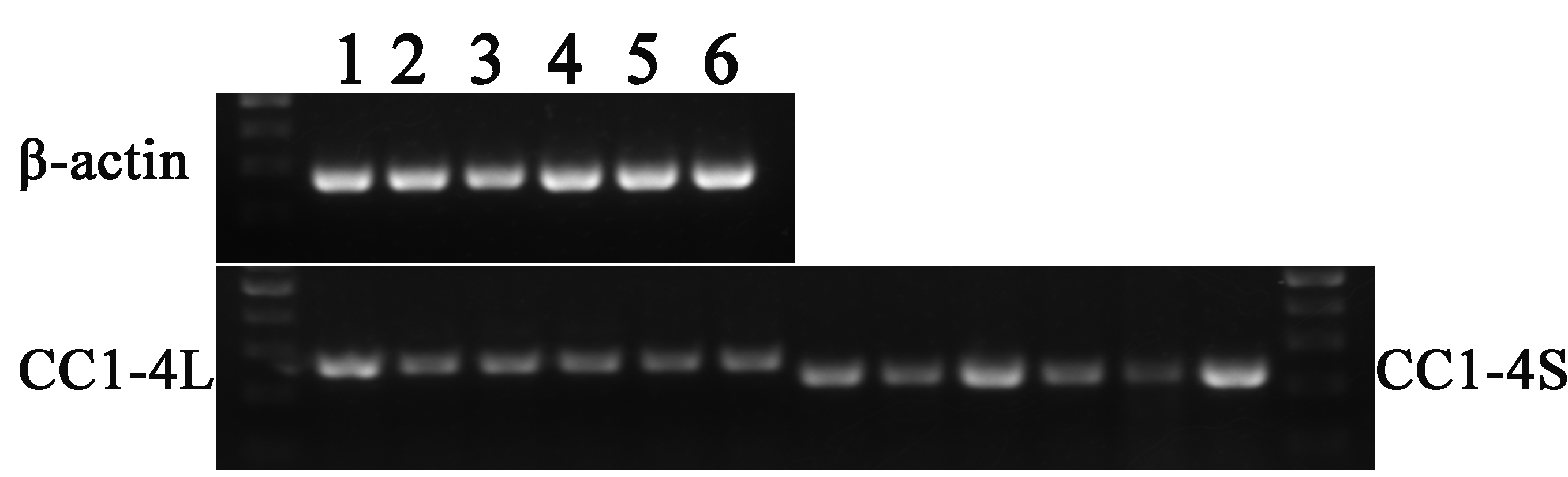

Supplement: Figure S1 — Semiquantitative RT-PCR analysis of CEACAM1-4L and CEACAM1-4L in TSCC tissues. The results showed that both CEACAM1-4L and CEACAM1-4S were moderately or strongly expressed in TSCC tissues. 1–6 represented 6 cases of the fresh TSCC tissues. (TIF) [file pone.0089991.s001.tif]

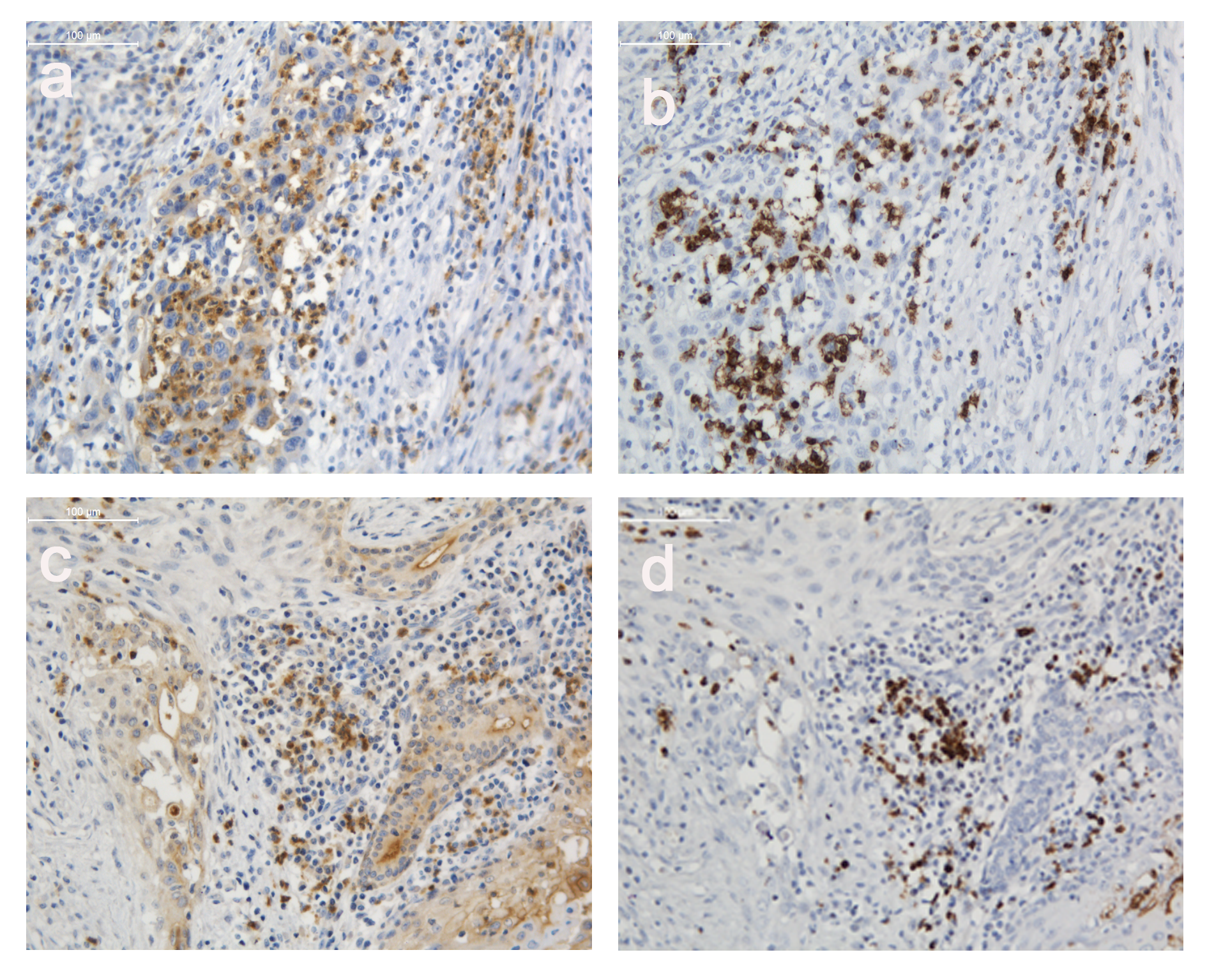

Supplement: Figure S2 — CEACAM1 and CD15 immunohistochemical staining for neutrophils in SCCOT tissues. The results showed that most of the CEACAM1 positive inflammatory cells were neutrophils and nearly all the neutrophils expressed strong CEACAM1. a, c: CEACAM1 staining; b, d: CD15 staining. (a, b, c, d 200×). (TIF) [file pone.0089991.s002.tif]
